# Editorial: Women in multiple sclerosis and other demyelinating disorders: A global perspective

**DOI:** 10.3389/fneur.2023.1148659

**Published:** 2023-02-21

**Authors:** Ethel Ciampi, Antonia Ceccarelli, Maria Isabel Zuluaga

**Affiliations:** ^1^Department of Neurology, Pontificia Universidad Católica de Chile, Santiago, Chile; ^2^Department of Neurology, Hospital Sotero Del Rio, Santiago, Chile; ^3^Department of Neurology, EpiCURA Centre Hospitalier, Ath, Belgium; ^4^Heart Rhythm Management Centre, Universitair Ziekenhuis Brussel - Vrije Universiteit Brussel, Brussel, Belgium; ^5^Multiple Sclerosis Center Medicarte, Medellin, Colombia

**Keywords:** multiple sclerosis, family planning, NMOSD, MOGAD, autoimmune encephalitis, global epidemiology

Our Research Topic aimed to review clinical and research studies focused on women with MS and other demyelinating diseases of the central nervous system (CNS), such as Aquaporin-4-IgG neuromyelitis optica spectrum disorder (NMOSD), myelin oligodendrocyte glycoprotein IgG-associated disease (MOGAD), and autoimmune encephalitis, with a global perspective.

Multiple sclerosis (MS) and other CNS demyelinating diseases are usually diagnosed in women. This accounts not only for sex-specific, hormonal, and pathophysiological differences but also for social factors, including family planning and employment. Moreover, many researchers, professors, and clinicians dedicated to MS are women, and global initiatives, such as the International Women in MS group, advocate for strategies supporting gender parity, diversity, and visibility. Sex-specific MRI differences in MS brain damage were investigated in a mini-review written by Ceccarelli. The article revealed that the majority of MRI studies seemed to confirm the existence of sex-related differences in brain structural and functional damage in MS, showing, despite some discrepancies, that women with MS seemed to have less neurodegenerative brain changes than men with MS. However, the author highlighted the need for future studies using large data sets, a longitudinal design, a machine learning approach, and correlations with hormonal changes, as well as a treatment response to elucidate further the role of sex in brain MS damage, since MS sex-based MRI studies are still scarce.

The majority of the articles submitted to our Research Topic explored different aspects of fertility and family planning in women with MS and other CNS demyelinating diseases. For instance, López-Reyes et al. reported the results of a survey on fertility preferences and unmet needs for family planning performed on 141 women living with MS in Bogotá, Colombia. They found that almost 50% of women did not desire to become pregnant in the future. Older age and a higher number of previous children were the only statistically significant factors related to this decision. Interestingly, when the women were questioned on their reasons for not wanting to become pregnant, 51% stated arguments related to their MS, including some misperceptions that could be clarified easily during clinical visits, particularly when deciding on disease-modifying treatments (DMTs) prescriptions; this shows that only 8% of women reported having discussed pregnancy with their neurologist prior to exposure to DMTs, thus emphasizing the physician's role in patient education. Moreover, the mini-review by Collorone et al. intended to shed light on the current knowledge of the protective role of the relapse rate of exclusive breastfeeding in MS and related ovarian suppression, semiologically associated with lactational amenorrhea, underscoring the potential immunomodulatory mechanisms related to this particular hormonal state during postpartum. Furthermore, this mini-review explored the use of DMTs during pregnancy and breastfeeding, combining FDA/EMA and manufacturers' advice with the postmarketing real-world evidence studies and biological properties of each DMT, which are moving our clinical practice toward a more active role in preventing postpartum relapses while also focusing on the infants' safety.

Women of childbearing age are also more prone to developing other antibody-mediated CNS disorders different than MS, such as NMOSD, MOGAD, and autoimmune encephalitis. Two articles on our Research Topic have, in turn, explored the impact of fertility, pregnancy, and employment on these diseases. In particular, the current knowledge regarding fertility, pregnancy, the postpartum period, treatment strategies, and the complex interplay between the immune system and these CNS diseases, as well as the need for future clinical research studies, were extensively analyzed by Cortese et al. in a must-read review. This review advocated for carefully planned care during the pregnancy and the postpartum period in these CNS diseases and highlighted the need for long-term prospective collaborative studies that at present are still lacking. On the other hand, NMOSD's impact on women's employment was investigated by Han et al.. The authors analyzed the current employment situation and economic burden as well as the risk factors for unemployment faced by women with NMSOD. The article revealed the enormous impact of illness on work, with more than half of participants reporting that the disease led to unemployment associated with older age, low educational level, higher annual relapse rate, and severe neurological disability. The medical expenses related to medication and hospitalization exerted a heavy financial burden on women patients with NMOSD.

Gender employment disparities in the field of MS neurology in Colombia were reported by Casallas-Vanegas et al.. Previous studies had reported that neurology is one of the fields in which female physicians are most underrepresented as first authors, and it also has one of the highest gender pay gaps. Casallas-Vanegas et al. showed that despite a higher number of female neurologists trained in MS, there are considerable gender gaps with regard to diverse opportunities at the academic, salary, promotion, leadership, teaching, and recognition levels between male and female MS neurologists.

Finally, the last article on this Research Topic proposed the first randomized controlled trial to compare two different neuromodulation techniques in the treatment of neurogenic bladder in women with MS in order to improve their quality of life. Çakir et al. developed a new single-center randomized controlled trial to compare two neuromodulation techniques [the posterior tibial nerve stimulation (PTNS) technique and repetitive transcranial magnetic stimulation (rTMS)] in the treatment of chronic neurogenic bladder in women with MS. Previous studies have already shown that these techniques improve bladder dysfunction and quality of life over pharmacological treatments in MS. However, no studies have directly compared the use of these two techniques on neurogenic bladder in women with MS.

In conclusion, the contributions received for this Research Topic covered: (1) sex-related structural and functional brain damage, (2) family planning, (3) gender disparities in patients living with CNS demyelinating diseases, and also in the neurologists treating those patients, and (4) new symptomatic treatment strategies aimed at improving the quality of life of MS patients, suggesting the need for further women-tailored research studies, treatment, and social strategies addressing MS and other CNS demyelinating diseases globally.

## Author contributions

EC, AC, and MZ contributed to the conception of the editorial, drafting the work or revising it critically for important intellectual content, providing approval for publication of the content, and agreeing to be accountable for all aspects of the work in ensuring that questions related to the accuracy or integrity of any part of the work are appropriately investigated and resolved. All authors contributed to the article and approved the submitted version.

